# An Open Label, Randomized, Comparative Bioavailability Study of BioTurm™ Extract Versus Curcuma longa Extract With Piperine in Healthy Adult Volunteers

**DOI:** 10.7759/cureus.97127

**Published:** 2025-11-17

**Authors:** Vineet K Malhotra, Anil C Deshpande, Sanjay Tamoli, Girish S Soman, Renuka Sabnis Shinde, Arati D Soman

**Affiliations:** 1 General Outpatient Department, Shivam Multispeciality and Accident Care Centre Pvt. Ltd., Pune, IND; 2 Department of Medical Education and Research, Target Institute of Medical Education and Research Pvt. Ltd., Mumbai, IND; 3 Department of Research and Development, Nisarga Biotech Pvt. Ltd., Satara, IND; 4 Department of Ayurved, Nisarga Biotech Pvt. Ltd., Satara, IND

**Keywords:** ar-turmerone, bioavailability, curcuma longa, curcuminoids, pharmacokinetics, piperine

## Abstract

Introduction

Curcumin (95%) from *Curcuma longa* rhizomes holds significant therapeutic promise, but its clinical efficacy is limited by poor oral bioavailability due to rapid metabolism, poor solubility, and quick elimination. This study compared the pharmacokinetics of BioTurm™, a novel standardized extract, with the market standards of *Curcuma longa* (95% curcumin) with piperine extract, a known bioavailability enhancer.

Methods

A single-dose, open-label, randomized, three-arm, comparative study was conducted in 14 healthy volunteers (aged 21-30 years). Participants were randomized into three groups: group A - BioTurm^TM^ extract (standardized to 45% curcuminoids and 4.5% ar-turmerone, n = 5), group B (95% curcuminoidswith 1% piperine, n = 4), and group C (95% curcuminoidswith 10% piperine, n = 5). Following single oral dose administration, blood samples were collected at multiple time points over eight hours. Plasma concentrations were analyzed using validated liquid chromatography-tandem mass spectrometry (LC-MS/MS), and pharmacokinetic (PK) parameters were determined.

Results

BioTurm™ (group A) achieved significantly higher bioavailability than market-standard group B, with a C_max_ of 647.97 ng/mL vs. 10.94 ng/mL. Group C, containing 10% piperine, achieved a comparable C_max _(663.60 ng/mL) and area under the curve (AUC₀-₂₄: 1479.89 ng·hr/mL). Ar-turmerone from BioTurm™ was detectable throughout the sampling period, peaking at two hours. No adverse events were reported.

Conclusion

BioTurm™ showed significantly enhanced bioavailability over the standard curcumin-piperine (1%) combination, without requiring additional enhancers. Only a high dose of piperine (10%) could match its PK profile. The presence of ar-turmerone and curcuminoids in BioTurm™ suggests a synergistic effect, offering a simplified and effective alternative to piperine-based formulations with potential therapeutic advantages.

## Introduction

*Curcuma longa* (turmeric) has garnered significant attention in the pharmaceutical and nutraceutical industries due to its well-documented therapeutic properties, particularly those attributed to its primary bioactive compound, curcumin [1]. Despite its promising pharmacological effects, including anti-inflammatory, antioxidant, and anticancer properties, curcumin's clinical efficacy is substantially limited by its poor oral bioavailability, primarily due to low solubility, rapid metabolism, and limited absorption in the gastrointestinal tract [1].

Recent advances in drug delivery systems and formulation technologies have focused on enhancing curcumin's bioavailability [2]. The traditional approach of combining *Curcuma longa* extract with piperine, a bioactive alkaloid from black pepper (*Piper nigrum*), has shown promise in improving curcumin absorption by inhibiting hepatic and intestinal glucuronidation [3,4]. However, the enhancement achieved through this conventional method may not be sufficient for optimal therapeutic outcomes. Moreover, relying on excess purified piperine can be costly and may cause gastrointestinal discomfort and interfere with drug metabolism, leading to potential drug interactions, as supported by safety and toxicological studies [5].

BioTurm™, a novel proprietary extract formulation, represents a significant advancement in addressing these bioavailability challenges. This innovative formulation employs a specialized extraction process and delivery system designed to overcome the physicochemical limitations of conventional curcumin extracts. BioTurm™ may offer superior pharmacokinetic properties due to its holistic nature, as compared to conventionally used pure curcumin and traditional *Curcuma longa* extracts, even when the latter is combined with piperine.

In addition to curcuminoids, ar-turmerone, a major bioactive constituent naturally present in turmeric oil, has been reported to enhance curcumin’s cellular uptake and modulate inflammatory signaling pathways, suggesting a possible synergistic role in improving its bioavailability and biological activity. This inherent synergism may partly explain the superior pharmacokinetic behavior observed with holistic turmeric matrix formulations like BioTurm™ [6,7].

Understanding the comparative bioavailability of these formulations is crucial for establishing evidence-based therapeutic protocols and optimizing clinical outcomes. While several studies have investigated various curcumin formulations [8], there is limited data directly comparing the bioavailability of BioTurm™ with conventional piperine-enhanced *Curcuma longa* extracts in human subjects under controlled conditions.

This study evaluated and compared the pharmacokinetic profiles of BioTurm™ extract versus standard *Curcuma longa *extract combined with piperine in healthy volunteers. It examined key parameters such as maximum plasma concentration (C_max_), time to reach maximum concentration (T_max_), area under the curve (AUC), and elimination half-life (t_1/2_), and established a comprehensive understanding of the relative bioavailability and absorption characteristics of these formulations.

We hypothesized that BioTurm™ would demonstrate superior oral bioavailability compared to a conventional *Curcuma longa *extract co-administered with piperine, owing to its specialized extraction process and the presence of naturally synergistic constituents such as ar-turmerone. The findings of this study contributed valuable insights to the growing body of research on curcumin bioavailability enhancement strategies and will have significant implications for the development of more effective curcumin-based therapeutic interventions.

## Materials and methods

Study design and ethics

This was a single-dose, open-label, randomized, three-arm, comparative bioavailability study conducted between October 2023 and February 2024. The study was conducted in accordance with Good Clinical Practice (GCP) and Good Laboratory Practice (GLP) guidelines, with approval from the Institutional Ethics Committee of Mhaske Hospital & Research Center Pvt. Ltd. (Gadital, Hadapsar, Pune), and was registered with the Clinical Trials Registry - India (CTRI/2023/10/058565). The study was conducted by an independent contract research organization.

The study design followed the PICO (Population-Intervention-Comparator-Outcome) framework to ensure methodological clarity. The population included healthy adult volunteers (male and female, 21-30 years). Intervention was a single oral dose of BioTurm™ extract standardized to 45% curcuminoids and 4.5% ar-turmerone. Comparators included conventional Curcuma longa extract (95% curcuminoids) with Piper longum extract (95% piperine) in 99:1 and 90:10 ratios. Outcome included comparative pharmacokinetic parameters (C_max_, T_max_, AUC₀_-t_, and t_½_) to evaluate relative oral bioavailability.

Study population

Fourteen healthy male and female participants aged 21-30 years were enrolled in the study. Subjects were included if they had no clinical or laboratory abnormalities based on preliminary hematological investigations (hemogram, liver function tests, renal function tests, and fasting blood sugar). Participants who consumed turmeric-containing products underwent a minimum 48-hour washout period before study initiation.

Test products and randomization

The BioTurm™ extract was provided by Nisarga Biotech Pvt. Ltd., Satara, India. Participants were randomized into three groups using a computer-generated randomization list. Group A (n = 5): BioTurm^TM^ extract standardized to 45% curcuminoids and 4.5% ar-turmerone. Group B (n = 4): Comparator I - *Curcuma longa* extract (95% curcuminoids) with *Piper longum* extract (95% piperine) in a 99:1 ratio. Group C (n = 5): Comparator II - *Curcuma longa* extract (95% curcuminoids) with *Piper longum* extract (95% piperine) in a 90:10 ratio.

CONSORT

**Figure 1:**
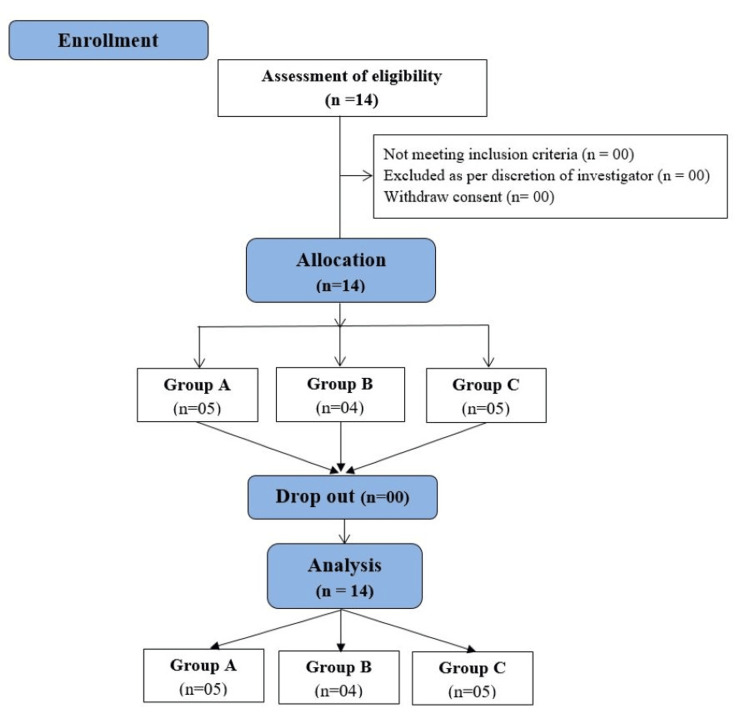
CONSORT diagram. CONSORT: Consolidated Standards of Reporting Trials.

The CONSORT (Consolidated Standards of Reporting Trials) flow diagram of the study is presented in Figure 1.

Statistical analysis

All pharmacokinetic parameters were compared between groups using one-way ANOVA. P-values were calculated separately for each parameter. All statistical analyses were performed by independent statisticians to reduce potential bias.

Sample size calculation

This study was designed as a pilot, exploratory investigation. Based on the clinical and scientific judgment of the investigators, a total of 14 healthy male and female participants were enrolled and completed the study (distributed as 5:4:5 across groups A, B, and C). Although the study was not powered for formal statistical inference, this sample size was considered adequate to generate preliminary pharmacokinetic data and identify trends across key parameters.

Pharmacokinetic blood sampling

Participants reported to the study site between 6 am and 8 am following a 10-hour fasting period. A baseline blood sample (2 mL) was collected via aseptic venipuncture from the cubital vein. Subjects then received a single oral dose of 8 g of their assigned study product with 150 mL of water. Blood samples (5 mL) were collected at 0, 0.5, 1, 2, 4, 5, 6, and 8 hours post-administration using an in-situ scalp vein set with a heparin lock. Blood samples were allowed to clot at room temperature for one hour, after which serum was separated and stored under appropriate conditions until analysis.

LC-MS/MS method development and bio-analysis

Serum samples were analyzed using liquid chromatography-tandem mass spectrometry (LC-MS/MS) (API 4000 Q trap mass spectrometer coupled with ExionLC System, SCIEX, Marlborough, MA) with ANALYST 1.6.1 software. Chromatographic separation was performed on a Venusil column (5μ, 4.6 × 100 mm) maintained at 40°C. The mobile phase consisted of (a) 5 mM ammonium acetate in Milli-Q water (40%) and (b) 0.1% formic acid in acetonitrile (60%), delivered at a 0.7 mL/minute flow rate. Sample processing involved protein precipitation using 0.1% formic acid in acetonitrile. The calibration curve range was 1.0-10000.0 ng/mL with a lower limit of quantification (LLOQ) of approximately 1.0 ng/mL.

Pharmacokinetic analysis

Pharmacokinetic parameters, including peak plasma concentration (C_max_), time to peak concentration (T_max_), and area under the plasma concentration-time curve (AUC_0-t_), were calculated using pk1 and pk2 software based on the plasma concentration-time profiles. The analysis was conducted at Dipon Ed Research International Pvt. Ltd., located in Bangalore, Karnataka, India.

## Results

Assessment of pharmacokinetic parameters

The mean peak plasma concentrations (C_max_) showed that BioTurm™ (group A, 647.97 ng/mL) demonstrated significantly higher bioavailability compared to the formulation with 1% piperine (group B, 10.94 ng/mL). The high-concentration piperine formulation (group C) achieved comparable concentrations (663.60 ng/mL) to BioTurm™.

The observed T_max_ was 4.0 hours for group A, slightly delayed at 4.50 hours for group B, and 4.0 hours for group C.

The area under the plasma concentration vs. time curve from time zero to 24 hours (AUC₀-₂₄) was highest in group C (1479.89 ng.hr/mL), followed closely by group A (1458.21 ng.hr/mL), while group B showed markedly lower exposure (25.13 ng.hr/mL).

The area under the plasma concentration vs. time curve extrapolated to infinity (AUC0-∞) demonstrated that BioTurm™ (group A, 1462.34 ng.hr/mL) achieved significantly higher bioavailability compared to the conventional 1% piperine formulation (group B, 30.93 ng.hr/mL). The 10% piperine formulation (group C, 1485.31 ng.hr/mL) was required to match BioTurm™ bioavailability.

**Figure 2:**
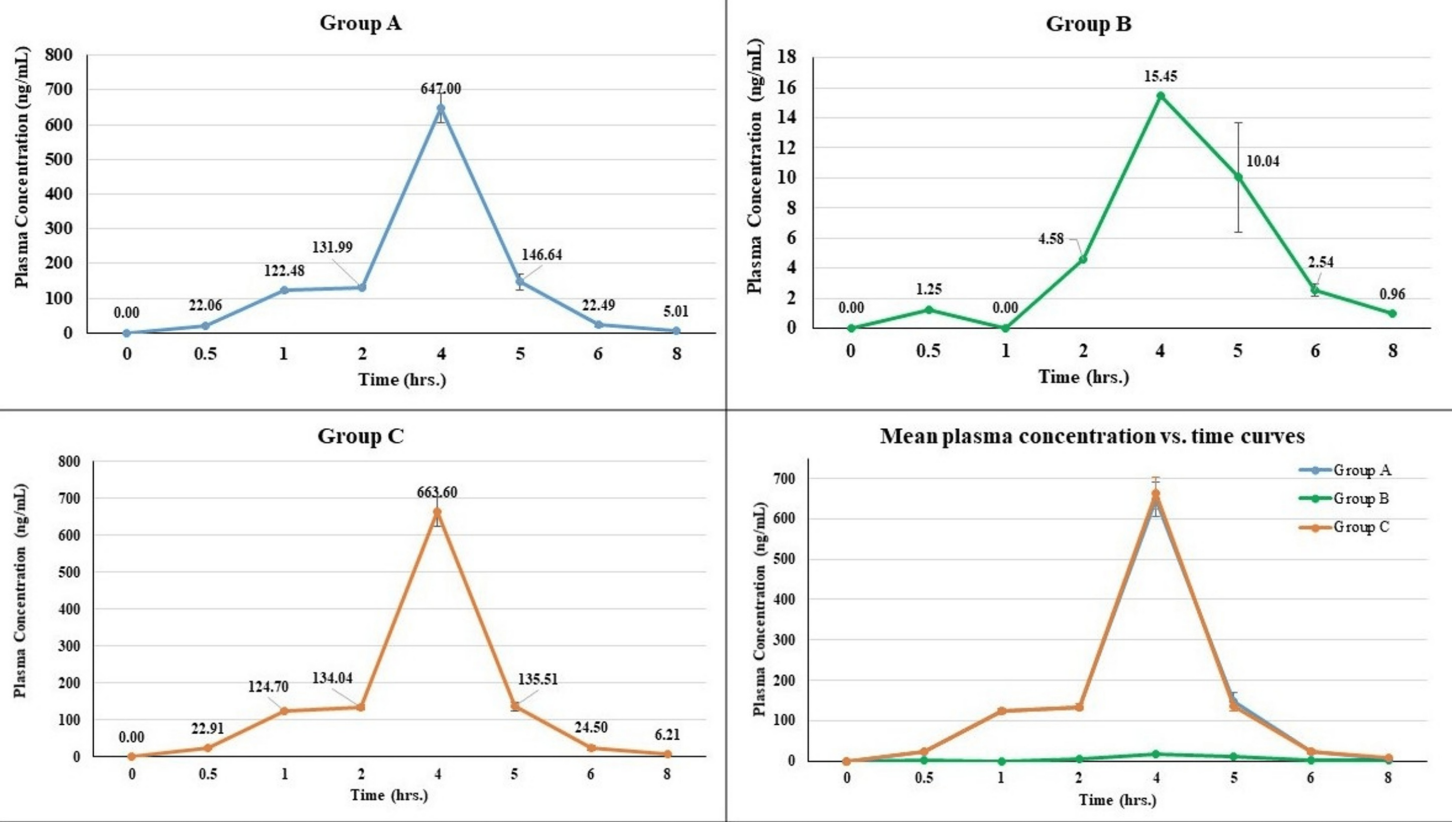
Comparative plasma concentration-time profiles showing enhanced bioavailability of BioTurm™ and 10% piperine formulation versus 1% piperine.

The elimination rate constant (K_el_) was similar for groups A and C (1.22 1/hr and 1.17 1/hr, respectively) but substantially lower for group B (0.45 1/hr). Correspondingly, the half-life (t_1/2_) was similar for groups A and C (0.57 hours and 0.59 hours, respectively) but notably longer for group B (3.26 hours) (Table 1 and Figure 2).

**Table 1:** Pharmacokinetic parameters for curcuminoid in the healthy human participants. Data were analyzed by the repeated factor, one-way ANOVA test. P < 0.05 was considered significant. PK: pharmacokinetic; AUC: area under the curve.

PK parameters	Group A	Group B	Group C	P-value between groups	F value
C_max _(ng/ml)	647.97 ± 42.59	10.94 ± 6.38	663.60 ± 40.87	0.0001	471.677
T _max_ (hr)	4 ± 0.00	4.50 ± 0.71	4 ± 0.00	0.801	2.289
K_el _(hr)	1.22 ± 0.05	0.45 ± 0.46	1.17 ± 0.09	0.01	11.347
t_1/2_ (h)	0.57 ± 0.02	3.26 ± 3.35	0.59 ± 0.04	0.0001	2.951
AUC_0-24 _(ng.hr/ml)	1458.21 ± 83.65	25.13 ± 28.92	1479.89 ± 92.77	0.0001	499.279
AUC_0-∞_ (ng.hr/ml)	1462.34 ± 83.65	30.93 ± 31.77	1485.31 ± 94.69	0.0001	482.323

Overall, the pharmacokinetic data demonstrate that BioTurm™(group A) showed significantly superior bioavailability compared to the market-standard 1% piperine formulation (group B), while requiring a substantially higher piperine concentration (10%, group C) to achieve comparable pharmacokinetic profiles.

Quantification of ar-turmerone using the LC-MS/MS method

The quantification of ar-turmerone in serum samples was performed using LC-MS/MS on an API 4000 Q trap mass spectrometer coupled with the Ultimate 300 LC system. Chromatographic separation was achieved using a Luna C18 column (4.6 × 150 mm, 3µ) maintained at 30°C, with an isocratic elution using 5 mM ammonium formate in Milli-Q water (pH 3.5) and acetonitrile (10:90) at a flow rate of 0.6 mL/minute. The LC-MS/MS detection was performed in positive electrospray ionization mode with multiple reaction monitoring (MRM) transition of m/z 217.1 > 119.1.

Plasma sample preparation involved protein precipitation using acetonitrile in a 1:4 ratio, followed by centrifugation at 4000 rpm for seven minutes.

The analysis of ar-turmerone concentration in group A revealed a peak mean concentration of 3.38 ng/mL at two hours post-administration. The plasma concentration-time profile showed initial detection at 0.5 hours (1.31 ng/mL), followed by fluctuating concentrations over the eight-hour sampling period, with notable levels at one hour (2.15 ng/mL), four hours (1.92 ng/mL), and six hours (2.23 ng/mL), before declining to 0.57 ng/mL at eight hours (Table 2).

**Table 2:** Ar-turmerone plasma samples mean concentrations (ng/mL) (Group A). Data are represented as mean ± SD.

Time points	Ar-turmerone (mean ± SD) (ng/mL)
0	0
0.5 (30 min)	1.31 ± 1.51
1	2.15 ± 2.47
2	3.38 ± 3.17
4	1.92 ± 1.79
5	0.83 ± 0.66
6	2.23 ± 2.95
8	0.57 ± 0.47

Adverse events

There were no adverse events or serious adverse events reported in any of the participants throughout the study period.

## Discussion

Turmeric (*Curcuma longa*), its extract, and various bioactive constituents have been extensively studied for their pharmacological benefits, metabolism, and safety. Among these, curcumin is the primary active compound known for its anti-inflammatory, antioxidant, and therapeutic potential. However, its clinical utility has been limited due to poor oral bioavailability, primarily attributed to rapid metabolism and systemic elimination [9].

To overcome these limitations, several strategies have been developed, including the use of bioavailability enhancers like piperine, liposomal delivery systems, and co-administration with turmeric essential oils. The current study evaluated the comparative bioavailability of curcuminoids from three formulations in healthy human volunteers, emphasizing the unique, holistic design of BioTurm™, a formulation standardized to both curcuminoids and ar-turmerone.

The pharmacokinetic evaluation demonstrated that BioTurm™ (group A) achieved significantly higher systemic exposure to curcuminoids compared to a conventional 95% curcumin formulation containing 1% piperine (group B), and comparable levels to a formulation with 10% piperine (group C). The mean peak plasma concentrations (C_max_) were 647.97 ng/mL for group A and 10.94 ng/mL for group B, while group C reached 663.60 ng/mL. Notably, both groups A and C reached maximum concentrations at four hours post-dose (T_max_ = 4 hours), suggesting similar absorption kinetics despite the absence of synthetic bioenhancers in BioTurm™. These findings align with previous reports on the limitations of curcumin absorption and the role of piperine in enhancing systemic levels [9-11].

Our results align with earlier human studies demonstrating that co-administration of curcumin with piperine markedly increases systemic exposure compared with curcumin alone. For example, one study reported a ~20-fold higher AUC in healthy volunteers receiving 2 g curcumin plus 20 mg piperine versus curcumin alone [11]. The comparable systemic exposure observed in the BioTurm™ group (group A) and the high-piperine group (group C) in our study likely reflects the formulation’s extraction and delivery design. The combination of curcuminoids and ar-turmerone may improve bioavailability by increasing solubility, facilitating membrane permeation, and reducing first-pass metabolism, thereby enabling effective delivery without reliance on enhancers. This rationale is consistent with current literature on curcumin bioavailability improvement strategies [12].

The enhanced performance of BioTurm™ may be attributed to the inclusion of ar-turmerone, a sesquiterpene component found in turmeric oil, which contributes not only pharmacological activity but also bioavailability enhancement [10]. Systemic exposure, as measured by AUC₀₋₂₄, further supports these findings: 1458.21 ng·hr/mL for group A, 25.13 ng·hr/mL for group B, and 1479.89 ng·hr/mL for group C. These results underscore the ability of BioTurm™ to deliver curcuminoids at levels comparable to high-dose piperine-enhanced products, without relying on synthetic enhancers, thereby preserving a more natural formulation profile [9-11].

In terms of elimination kinetics, groups A and C demonstrated similar elimination rate constants (1.22 and 1.17 1/hr, respectively) and half-lives (0.57 and 0.59 hours), while group B exhibited a slower elimination (K_el_: 0.45 1/hr) and a longer half-life (3.26 hours). These differences may result from altered curcuminoid metabolism and slower systemic uptake in the absence of effective enhancers [10,11].

Crucially, the study also measured plasma levels of ar-turmerone in group A to determine its absorption and potential contribution to curcuminoid bioavailability. Detectable plasma concentrations were observed for eight hours post-administration, peaking at two hours (3.38 ng/mL). This confirms systemic absorption of ar-turmerone and supports its hypothesized role in enhancing intestinal transport and reducing efflux via modulation of P-glycoprotein activity [7]. These mechanisms have previously been demonstrated in Caco-2 cell models and animal studies [7,13].

Beyond its bioavailability-enhancing effects, ar-turmerone itself possesses multiple pharmacological properties, including anti-inflammatory, hepatoprotective, antimicrobial, antifungal, and antitumor effects [10]. In particular, its ability to induce apoptosis in hepatocellular carcinoma cells via reactive oxygen species (ROS)-mediated pathways has been well documented, supporting its broader therapeutic potential within a curcuminoid-based formulation [10].

The holistic approach of BioTurm™, which preserves both major phytoconstituents, i.e., curcuminoids and ar-turmerone, offers a composition that reflects the natural synergy found in whole turmeric. This aligns with the growing interest in phytocomplexes that maintain the full spectrum of a plant’s bioactives rather than relying on isolated compounds or synthetic additives [7,13,14]. The ability to achieve clinically relevant plasma levels through this naturally balanced extract may offer both safety and efficacy advantages, particularly for long-term use.

From a safety perspective, all formulations were well tolerated, with no adverse events or serious adverse events reported in any group during the study period. This finding is consistent with earlier human trials involving curcumin and turmeric extracts [14].

In summary, the results of this study provide strong evidence for the superior bioavailability of BioTurm™ without the need for synthetic enhancers like piperine. The inclusion of ar-turmerone appears to play a dual role of enhancing absorption while also contributing pharmacological benefits. These findings support the use of optimized whole extracts of *Curcuma longa* in clinical settings and provide a compelling rationale for future studies to further explore the long-term efficacy, safety, and mechanistic synergy between curcuminoids and ar-turmerone.

Detection of ar-turmerone in plasma is a novel and noteworthy finding in this study; its proposed role in modulating intestinal transporters such as P-glycoprotein remains a mechanistic hypothesis and was not directly evaluated here. Therefore, we present this as a potential contributor to the enhanced bioavailability of BioTurm™, rather than a confirmed pathway.

The present study included both male and female volunteers; however, the small sample size and inherent inter-individual variability may limit generalizability. The short, single-dose design of the study may not reflect long-term effects or applicability across diverse populations. Future studies with larger cohorts and extended durations are needed to further validate these findings and explore population-level pharmacokinetic diversity and clinical relevance.

## Conclusions

The pharmacokinetic analysis demonstrates that BioTurm™, combining curcuminoids and ar-turmerone, provides superior bioavailability compared to low-dose piperine (1%) formulations and comparable exposure to high-dose piperine (10%), without the need for external bioenhancers. The findings suggest a synergistic role of ar-turmerone in enhancing curcumin absorption, supporting the value of a natural, whole-extract approach that aligns with clean-label product development and long-term safety considerations. Future studies with larger cohorts and multi-dose designs are needed to further substantiate these results and evaluate their broader clinical applicability in therapeutic and preventive health applications.
